# A Polymorphism in the Splice Donor Site of ZNF419 Results in the Novel Renal Cell Carcinoma-Associated Minor Histocompatibility Antigen ZAPHIR

**DOI:** 10.1371/journal.pone.0021699

**Published:** 2011-06-28

**Authors:** Kelly Broen, Henriette Levenga, Johanna Vos, Kees van Bergen, Hanny Fredrix, Annelies Greupink-Draaisma, Michel Kester, J. H. Frederik Falkenburg, Pieter de Mulder, Theo de Witte, Marieke Griffioen, Harry Dolstra

**Affiliations:** 1 Department of Laboratory Medicine – Laboratory of Hematology, Radboud University Nijmegen Medical Centre, Nijmegen, The Netherlands; 2 Department of Hematology, Radboud University Nijmegen Medical Centre, Nijmegen, The Netherlands; 3 Department of Hematology, Leiden University Medical Center, Leiden, The Netherlands; 4 Department of Medical Oncology, Radboud University Nijmegen Medical Centre, Nijmegen, The Netherlands; 5 Department of Tumor Immunology, Nijmegen Centre for Molecular Life Sciences, Radboud University Nijmegen Medical Centre, Nijmegen, The Netherlands; Institut national de la santé et de la recherche médicale (INSERM), France

## Abstract

Nonmyeloablative allogeneic stem cell transplantation (SCT) can induce remission in patients with renal cell carcinoma (RCC), but this graft-versus-tumor (GVT) effect is often accompanied by graft-versus-host disease (GVHD). Here, we evaluated minor histocompatibility antigen (MiHA)-specific T cell responses in two patients with metastatic RCC who were treated with reduced-intensity conditioning SCT followed by donor lymphocyte infusion (DLI). One patient had stable disease and emergence of SMCY.A2-specific CD8+ T cells was observed after DLI with the potential of targeting SMCY-expressing RCC tumor cells. The second patient experienced partial regression of lung metastases from whom we isolated a MiHA-specific CTL clone with the capability of targeting RCC cell lines. Whole genome association scanning revealed that this CTL recognizes a novel HLA-B7-restricted MiHA, designated ZAPHIR, resulting from a polymorphism in the splice donor site of the ZNF419 gene. Tetramer analysis showed that emergence of ZAPHIR-specific CD8+ T cells in peripheral blood occurred in the absence of GVHD. Furthermore, the expression of ZAPHIR in solid tumor cell lines indicates the involvement of ZAPHIR-specific CD8+ T cell responses in selective GVT immunity. These findings illustrate that the ZNF419-encoded MiHA ZAPHIR is an attractive target for specific immunotherapy after allogeneic SCT.

## Introduction

Allogeneic stem cell transplantation (SCT) has become the treatment of choice for patients with various hematological malignancies and some studies have also shown that metastatic renal cell carcinoma (RCC) does respond to this therapy [1]. Several studies have explored allogeneic SCT after nonmyeloablative or reduced intensity conditioning (RIC) with or without donor lymphocyte infusions (DLI) as curative treatment for metastatic RCC, and objective response rates varied from 0% to 53% [1]–[6]. However, substantial transplantation-related mortality and toxicity due to graft-versus-host-disease (GVHD) has been observed. Therefore, further development of allogeneic SCT for solid tumors demands a more specific approach to selectively boost graft-versus-tumor (GVT) reactivity without enhancement of GVHD.

Minor histocompatibility antigens (MiHA) are the target antigens of the GVT response, and expansion of MiHA-specific cytotoxic T lymphocytes (CTL) usually precedes clinical remission of the malignancy in patients treated with DLI [7]–[9]. However, alloreactive CTL responses induced upon DLI generally lack tumor specificity and are often accompanied by GVHD. Therefore, it would be highly beneficial to direct T cell immunity towards MiHA that are selectively expressed on malignant cells. Only a few tissue-restricted MiHA have been described that are aberrantly expressed on solid tumors, including HA-1, ECGF-1, BCL2A1, LRH-1 and C19orf48 [10]–[14]. In addition to a MiHA, an immunogenic antigen has been described expressed on metastatic RCC, HERV-E which is derived from a human endogenous retrovirus [15]. However, further characterization of the target antigens of alloreactive T cells on RCC tumor cells is of great importance for the development of specific post-transplant immunotherapy for metastatic RCC.

Here, we characterized MiHA-specific T cell responses in two patients with metastatic RCC treated with partial T cell-depleted RIC-SCT followed by DLI. Presence of MiHA-specific CTL targeting RCC tumor cells *in vitro* could be demonstrated in both patients who experienced partial regression or stable disease following RIC-SCT and DLI without clinical evidence of GVHD. In one patient the T cell response was directed against the HLA-A2-restricted SCMY peptide FIDSYICQV, and in the other patient a MiHA-specific CTL clone emerged with the capability of targeting RCC cell lines. We found that this CTL recognizes an HLA-B7-restricted MiHA resulting from a polymorphism in the splice donor site within the ZNF419 gene. This novel MiHA, designated ZNF419 alternatively spliced polymorphic histocompatibility antigen in RCC (ZAPHIR), is co-expressed by RCC and transformed B cells, but is not presented by non-hematopoietic fibroblasts. Furthermore, ZAPHIR-specific CD8+ T cells were detectable in peripheral blood post-transplant using tetramer analysis. These findings exemplify that ZAPHIR represents a new target antigen for the development of adjuvant immunotherapy for RCC and hematological malignancies after allogeneic SCT.

## Results

### Assessing for alloreactive MiHA-specific CD8+ CTL after transplantation of metastatic RCC patients

**Figure 1:**
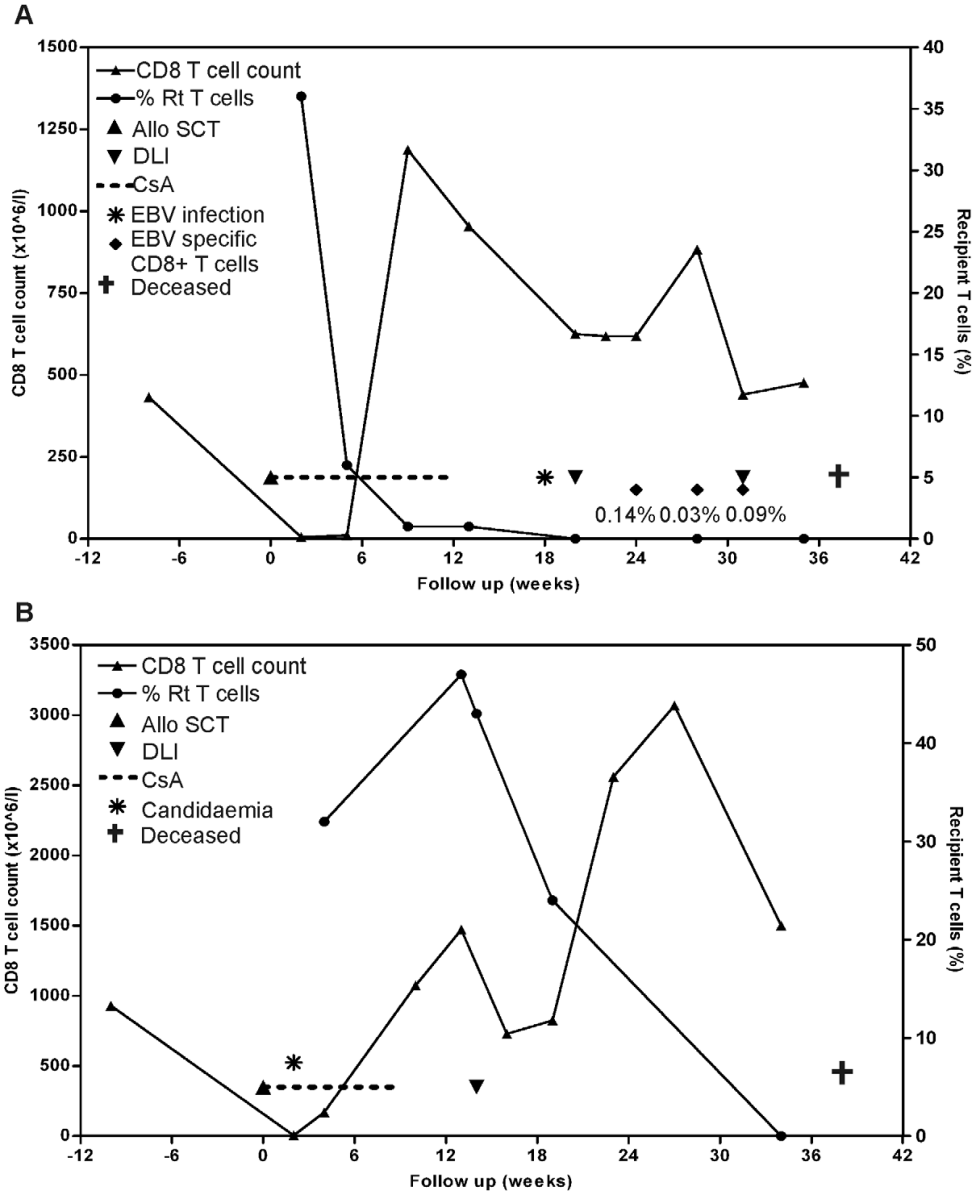
Longitudinal follow-up of CD8+ T cells in peripheral blood from metastatic RCC patients UPN677 and UPN686 in relation to clinical outcome. (**A**) UPN677: percentages of recipient T cells (right y axis) are compared with CD8+ T cell count ×10^6^ per liter peripheral blood (left y axis). Administration of SCT and DLI 1–2 are indicated by ▴ and ▾, respectively. Treatment interval with CsA is shown by the dotted line. Time points of confirmed EBV infection, occurrence of EBV-specific T cells (depicted as % from total CD8+ T cell population) and death are indicated. (**B**) UPN686: for legend see (A). Time points of confirmed candidemia and death are indicated.

Four patients with progressive metastatic RCC after treatment with at least one line of therapy were treated with RIC-SCT from an HLA identical sibling donor (Table 1). These patients had undergone tumor nefrectomy at a median of 4.9 years (range 2.8–11.1 years) before transplantation. After RIC-SCT, all patients reached eventually complete donor chimerism in the T cell fraction. The first tumor evaluation in UPN677 at three months after RIC-SCT showed stable disease, paralleled by expansion of CD8+ T cells during tapering of cyclosporine A (CsA). One month later this patient developed encephalopathy attributed to EBV-reactivation. Following DLI, a second expansion of CD8+ T cells as well as circulating EBV-specific CD8+ T cells was detected up to 11 weeks using tetramers against the GLCTLVAML epitope. At seven months after RIC-SCT a second DLI was administered and the tumor in the abdomen remained stable without occurrence of GVHD (Figure 1A). Evaluation of disease response in UPN686 3 months after RIC-SCT showed partial regression of pulmonary metastases that coincided with expansion of CD8+ T cells during tapering of CsA and before the first DLI. This patient received DLI at 3 months after RIC-SCT inducing a second CD8+ T cell expansion and conversion to complete donor T cell chimerism (Figure 1B). Three months later (*i.e.* 6 months after RIC-SCT) CT-scan evaluation showed stable disease. Unfortunately, this patient died from invasive fungal infection 9 months after RIC-SCT. Post-mortal examination showed multiple histological-confirmed fungal lesions and two pulmonary metastasis were found, but no signs of GVHD. Collectively, the chimerism kinetics and expansions of CD8+ T cells in UPN677 and UPN686 indicated T cell alloreactivity, but episodes with infections may have interfered.

**Table 1:** Patient characteristics, GVHD, DLI and outcome.

Patient	UPN651	UPN677	UPN705	UPN686
Age/Sex	57/M	53/M	60/M	49/M
Histology	Clear cell	Papillary	Clear cell	Clear cell
Metastases	Lung and mediastinum	Soft tissue	Lung, lymph node, thyroid, soft tissue	Lung
Previous therapy	IFN-α	IL-2, G250 mAb and RT (2 courses)	IFN-α with 6 Retinoic acid and resection of metastases	IL-2, IFN-α, 5FU (2 courses) and DC vaccination
GVHD acute	no	no	Grade II	no
GVHD chronic	limited	no	na	no
DLI(x10^8^T cells/kg)	no	0.1 and 0.7	no	0.1
Outcome	PD, died from meningo-encephalitis during dexamethasone (5 months)	SD, died from CMV-pneumonitis (8.5 months)	ne, died from multi-organ failure (1.3 months)	PR, died from invasive fungal infection (8.8 months)

Abbreviations: IFN-α, interferon-α; IL-2, interleukin-2, G250 mAb, monoclonal antibody against G250; RT, radiotherapy; 5FU, 5-fluorouracil; GVHD, graft-versus-host-disease; DLI, donor lymphocyte infusion; PD, progressive disease, SD stable disease; na, not applicable; ne, not evaluated; PR partial remission.

To investigate whether MiHA-specific CD8+ T cells targeting RCC tumor cells could be isolated from these two patients post DLI-1, IFN-γ+ CD8+ T cells (≈2%) were sorted and weekly restimulated with irradiated recipient peripheral blood mononuclear cells (PBMC) obtained pre-SCT. The resulting CD8+ T cell lines, termed CTL line H (UPN677) and CTL line B (UPN686), displayed significant IFN-γ production against recipient EBV-LCL, but not towards donor EBV-LCL, indicating the recognition of a disparate MiHA (Figure S1). These data indicate that MiHA-specific CD8+ T cells were present *in vivo* in RCC patients UPN677 and UPN686 following allogeneic RIC-SCT and DLI.

### Emergence of SMCY-specific CTLs in a RCC patient after DLI

**Figure 2:**
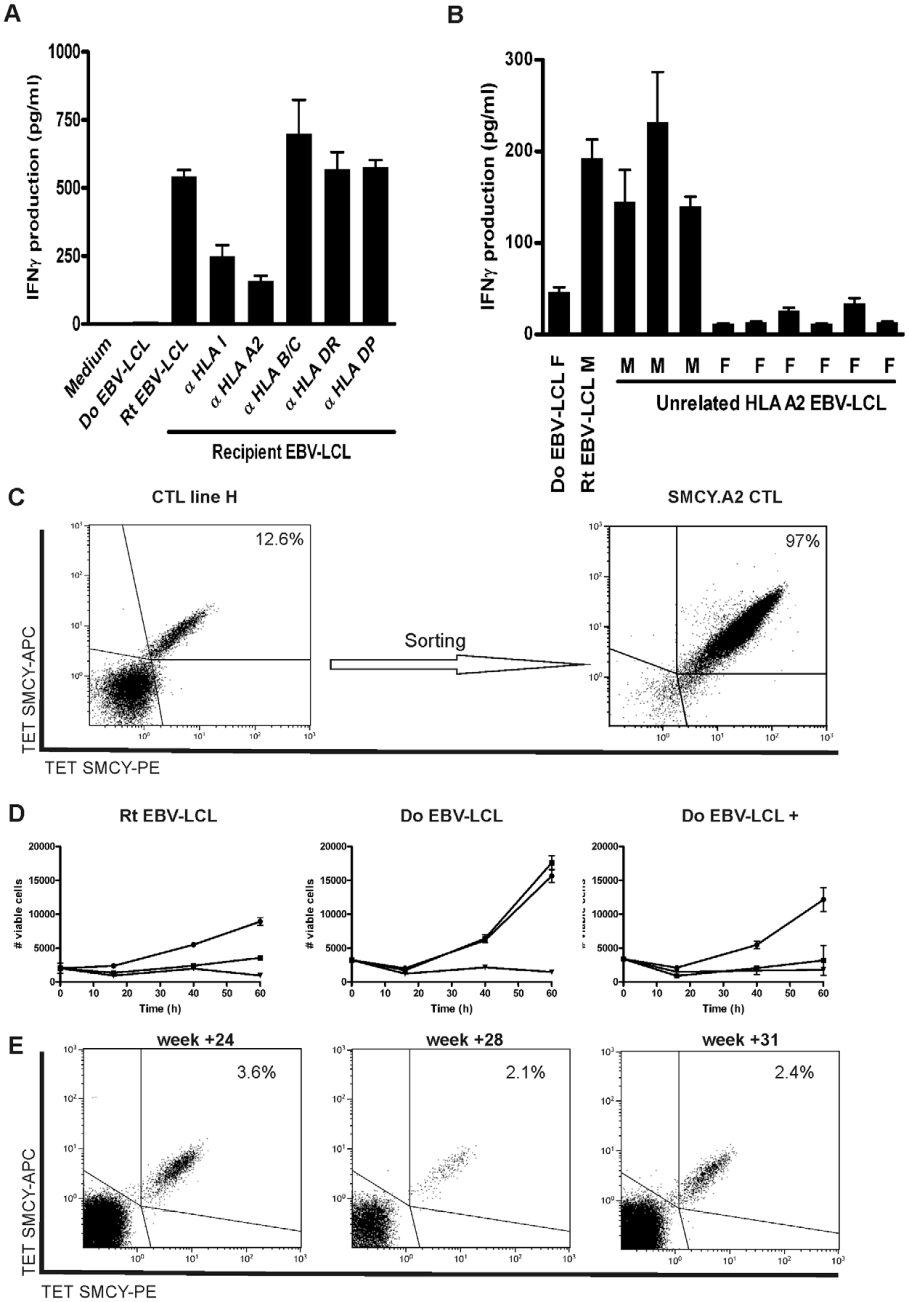
CD8+ T cells reactive with the SMCY.A2 peptide developed after DLI in RCC patient UPN677. (**A**) HLA-restriction was determined by the production of IFN-γ released by CTL line H upon stimulation with recipient EBV-LCL in the presence of HLA blocking antibodies. Data are displayed as mean IFN-γ release ± SD of triplicate wells. (**B**) Production of IFN-γ by CTL line H stimulated with EBV-LCL of 9 HLA-A2+ unrelated individuals, showing recognition of 3 out of 9 EBV-LCL of male origin. (**C**) Flow cytometry analysis of CD8+ CTL line H simultaneously stained PE- and APC-conjugated SMCY.A2 tetramer, CD8 AlexaFluor 700, CD4, CD14, CD16 and CD19 FITC and Sytox Blue. CD8+ T cells were gated on FITC- and Sytox Blue- cells. The percentage of SMCY.A2 tetramer-binding cells among viable CD8+ T cells was 12.6%. These SMCY.A2 tetramer+ CD8+ T cells were sorted and expanded resulting in a >95% pure population. (**D**) Survival of the recipient and donor EBV-LCL, and donor EBV-LCL pulsed with peptide FIDSYICQV was determined in a flow cytometry-based cytotoxicity assay after incubation with SMCY.A2-specific CTL from patient UPN677 (▪), allo-HLA-A2 CTL (▾; positive control) or medium only (•) at an E:T ratio of 3∶1 in the presence of 25 U/ml IL-2. Data are depicted as mean ±SD of triplicate wells. (**E**) Detection of SMCY.A2-specific CD8+ T cells in peripheral blood of RCC patient UPN677. PBMC collected 24, 28 and 31 weeks post DLI-1 were simultaneously stained with PE- and APC-conjugated SMCY.A2 tetramer, CD8 AlexaFluor 700, CD4, CD14, CD16 and CD19 FITC and Sytox Blue. Subsequently, cell populations were analyzed by flow cytometry. Cells were gated on CD8+FITC-Sytox Blue- lymphocytes, and the percentage of tetramer-binding cells among CD8+ T cells is depicted.

Next, we revealed TCR-Vb1+CD8+ cells to be the dominant T cell population in CTL line H using TCR-Vb PCR and flow cytometry (data not shown). Functional analysis showed that specific IFN-γ production by CTL line H was substantially inhibited by anti-HLA class I and anti-HLA-A2 antibodies, but not by antibodies against anti-HLA-B/C and anti-HLA class II (Figure 2A). Furthermore, testing EBV-LCL from unrelated HLA-A2+ individuals revealed that 3 out of 9 individuals were recognized, which were all of male origin (Figure 2B). These observations suggested that the dominant TCR-Vb1+CD8+ T cells in CTL line H recognize an HLA-A2-restricted HY antigen. Therefore, we stained CTL line H with PE- and APC-conjugated tetramers for HLA-A2-restricted SMCY.A2 epitope FIDSYICQV and found around 12.5% tetramer+ CD8+ T cells (Figure 2C). These SMCY.A2 tetramer+ T cells confirmed to be TCR-Vb1+ by flow cytometry (data not shown). A greater than 95% pure population of SMCY.A2 tetramer+ CTLs was isolated by flow cytometry allowing further characterization of its cytotoxic potential (Figure 2C). Flow cytometry-based cytotoxicity assays revealed that the TCR-Vb1+ SMCY.A2 CTL induced high levels of cytotoxicity against HLA-A2+ peptide-loaded SMCY- donor EBV-LCL as well as SMCY+ recipient EBV-LCL (Figure 2D). Tetramer analysis showed that SMCY.A2-specific CD8+ T cells became detectable *in vivo* in the post-DLI setting, constituting 3.6%, 2.1% and 2.4% of the CD3+CD8+ T cell population collected at week +24, +28 and +31, respectively (Figure 2E). Collectively, these data show that SMCY.A2 CD8+ T cells expanded in RCC patient UPN677 after DLI with the potential of targeting SMCY-expressing RCC tumor cells in the absence of GVHD.

### Identification of a novel HLA-B7-restricted MiHA resulting from a polymorphism in the splice donor site of ZNF419

**Figure 3:**
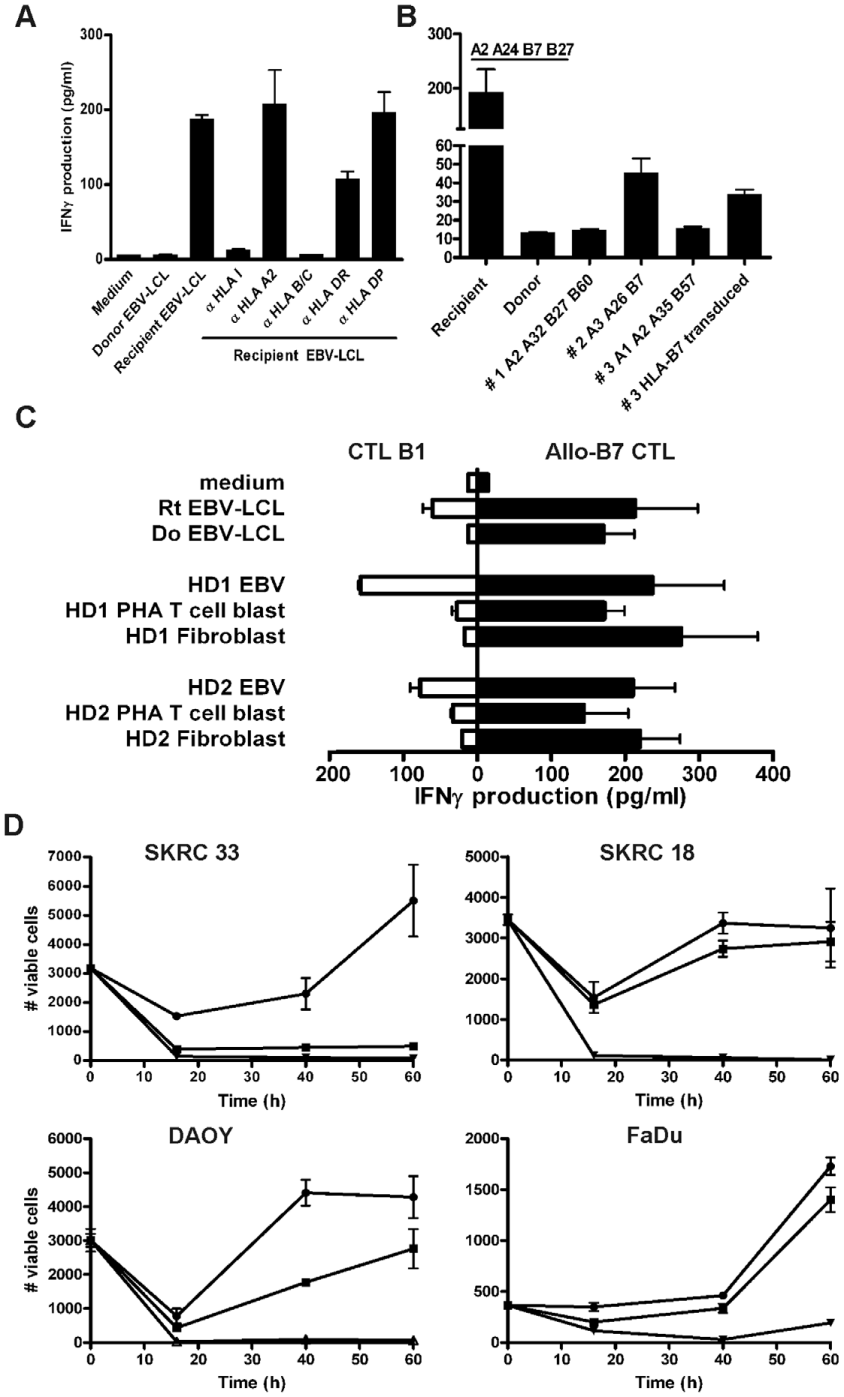
Cytotoxicity of HLA-B7-restricted CTL B1 against RCC and hematological tumor cell lines. (**A**) HLA-restriction was determined by the production of IFN-γ released by CTL B1 upon stimulation with recipient EBV-LCL in the presence of HLA blocking antibodies. (**B**) Production of IFN-γ by CTL B1 stimulated with rt EBV-LCL, EBV-LCL of an unrelated individual (#2) sharing HLA-B7 with the recipient, and an EBV-LCL of an HLA class I-mismatched individual (#3) that was transduced with HLA-B*0702. (**C**) Production of IFN-γ by CTL B1 and allo-HLA-B7 CTL stimulated with MiHA+ EBV-LCL and PHA-stimulated T cells and bone marrow-derived fibroblasts. Fibroblasts were pretreated with 10 ng/ml TNFα and 200 U/ml IFNγ for 2 days before coculture with CTL B1 (E:T ratio 10∶1). (**D**) MiHA mediated cytotoxicity on the HLA-B7+ RCC cell lines SKRC33 (rs2074071 genotype AA) and SKRC18 (rs2074071 genotype GG), the brain tumor cell line DAOY (rs2074071 genotype AG), and pharynx cell line FaDu (rs2074071 genotype GG), in flow cytometry-based cytotoxicity assays was determined after incubation with CTL B1 (▪), allo-HLA-B7 CTL (▾; positive control) or medium only (•) at an E:T ratio of 3∶1 in the presence of 25 U/ml IL-2. Data are depicted as mean ±SD of triplicate wells.

TCR receptor analysis using PCR and flow cytometry showed a predominant TCR-Vb4+CD8+ population in CTL line B, which were sorted and cultured resulting in a pure population (data not shown). This TCR-Vb4+CD8+ CTL, designated CTL B1, mediated specific IFN-γ production against recipient EBV-LCL, but not towards donor EBV-LCL (Figure 3A). Release of IFN-γ could be completely inhibited by anti-HLA class I and anti-HLA-B/C antibodies, but not by antibodies against anti-HLA-A2 and anti-HLA class II (Figure 3A). Testing of EBV-LCL from unrelated individuals sharing expression of HLA-B7 with the recipient, and EBV-LCL from an HLA class I-mismatched individual that were transduced with HLA-B*0702, revealed that CTL B1 recognizes an HLA-B7-restricted MiHA (Figure 3B).

Next, we investigated whether the MiHA recognized by CTL B1 was selectively expressed by hematopoietic cells. Therefore, EBV-LCL, PHA-stimulated T cells and non-hematopoietic fibroblasts from two MiHA+ HLA-B7+ healthy donors were used as target cells. CTL B1 produced IFN-γ upon co-culture with EBV-LCL, whereas PHA-stimulated T cells and TNF-α/IFN-γ pre-treated bone marrow-derived fibroblasts were not recognized (Figure 3C). In contrast, all target cell types were significantly recognized by allo-HLA-B7 CTL, indicating their susceptibility to CTL-mediated recognition (Figure 3C). To investigate whether the MiHA targeted by CTL B1 is expressed by RCC tumor cells, we performed flow cytometry-based cytotoxicity assay. We observed that CTL B1 significantly targets the HLA-B7+ RCC cell line SKRC33 and not SKRC18, showing MiHA mediated cytotoxicity (Figure 3D). In addition to these RCC cell lines, CTL B1 also recognizes and kills other solid tumor cell lines such as the HLA-B7+ brain cancer cell line DAOY, but not pharynx cell line FaDu. Allo-HLA-B7-specific CTL efficiently lysed all solid tumor cell lines tested (Figure 3D).

**Figure 4:**
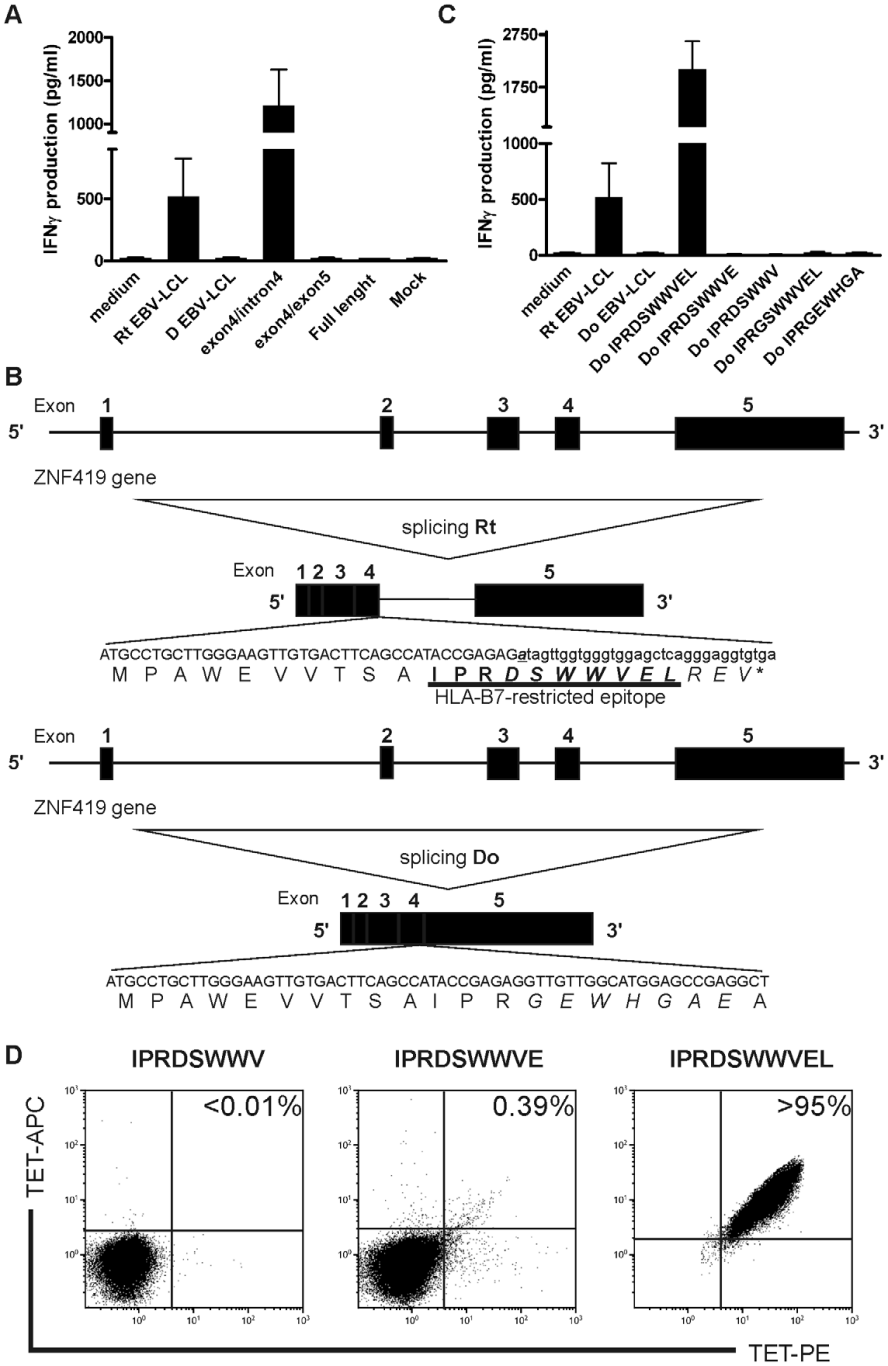
A SNP in the splice donor site between exon 4 and 5 of the ZNF419 gene determines CTL B1 recognition. (**A**) IFN-γ production by CTL B1 upon stimulation with 293T-HLA-B*0702/ICAM/CD80 cells transfected with ZNF419 full length, cloned splice variant containing the exon4/exon5 boundary and the alternative ZNF419 splice variant containing intron 4. (**B**) Schematic representation of the ZNF419 gene on chromosome 19q13.43. The nucleotide and deduced amino acid sequences of the alternatively and correctly spliced transcripts present in recipient and donor, respectively, are shown. Disparity between the recipient and donor ZNF419 protein sequence is due to a G to A polymorphism in de splice donor site of intron 4 leading to the ZAPHIR epitope (underlined). (**C**) ZAPHIR epitope reconstitution with synthetic peptides corresponding to HLA-B7 10-mer peptide IPRDSWWVEL, 9-mer peptide IPRDSWWVE, 8-mer peptide IPRDSWWV, HLA-B7 10-mer peptide IPRGSWWVEL and HLA-B7 9-mer peptide IPRGEWHGA. Donor EBV-LCL was pulsed with 10 µM peptide and tested for recognition by CTL B1 in the presence of 25 U/ml IL-2. Data are displayed as mean ± SD of triplicate wells. (**D**) Flow cytometry analysis of CTL B1 with tetramers containing the 8-, 9-, or 10-mer peptide. Cells were stained with PE- and ACP-conjugated tetramer, CD8 AlexaFluor 700, CD4 FITC and Sytox Blue. Cells were analyzed by flow cytometry by gating on CD8+CD4-Sytox Blue- lymphocytes. The percentage of tetramer+ cells among CD8+ T cells is depicted.

To map the chromosomal region containing the gene encoding the MiHA recognized by CTL B1, we performed whole genome association scanning (WGAs). A total of 87 endogenous HLA-B7+ EBV-LCL were tested and 42 were recognized by CTL B1 (Figure S2A). Comparing CTL recognition with SNP genotypes showed a strong association for a cluster of 5 SNPs on chromosome 19 (Figure S2B). This genetic region included the ZNF419 gene which is located at 19q13.43. There are 7 different splice variants of the ZNF419 gene (Figure S3A-B), from which several are expressed by patient UPN686 and its related donor (Figure S3C). To confirm that the MiHA is encoded by the ZNF419 gene, we cloned the different splice variants of the ZNF419 gene from both the donor and recipient EBV-LCL. The various constructs were transiently transfected into 293T-HLA-B*0702/ICAM/CD80 cells and tested for CTL B1 recognition. CTL B1 efficiently recognized cells transfected with a construct encoding an alternative ZNF419 isoform of the MiHA-positive recipient EBV-LCL (Figure 4A), whereas none of the ZNF419 isoforms cloned from the donor EBV-LCL stimulated IFN-γ production by CTL B1 (data not shown). Sequence analysis revealed that the recognized ZNF419 isoform results from the A/G SNP rs2074071 located in the splice donor site involved in the joining of exon 4 to intron 4. As a result of the G to A substitution in the splice donor site, the alternative ZNF419 transcript of the recipient contains a disparate amino acid sequence compared with that of the donor. By analyzing this disparate sequence for HLA-B7 binding peptides using the NetMHC server, we identified the strong HLA-B7 binding peptide IPRDSWWVEL that is located over the exon4-intron4 boundary (Figure 4B). We synthesized this 10-mer IPRDSWWVEL peptide (encoded by rs2074071 A variant), the 9-mer IPRDSWWVE peptide and the 8-mer IPRDSWWVEL peptide. We tested the HLA-B7 decamer peptide IPRGSWWVEL that would be encoded by the G genotype as well. In addition, we synthesized the HLA-B7 9-mer peptide IPRGEWHGA that is located on the exon4-exon5 boundary of the full length ZNF419 transcript. Their ability to stimulate IFN-γ release by CTL B1 upon loading of synthetic peptide on donor EBV-LCL was tested and only the IPRDSWWVEL epitope was recognized (Figure 4C). Flow cytometry analysis of CTL B1 showed that only the tetramer containing the 10-mer peptide was positive as opposed to tetramers containing the 8-, or 9-mer peptide (Figure 4D). These data demonstrate that the 10-mer peptide IPRDSWWVEL is the naturally presented epitope that is recognized by CTL B1. Based on these findings, we designated this novel MiHA as ZAPHIR for ZNF419 alternatively spliced polymorphic histocompatibility antigen in RCC, which is the result of a polymorphism in a splice donor site.

**Figure 5:**
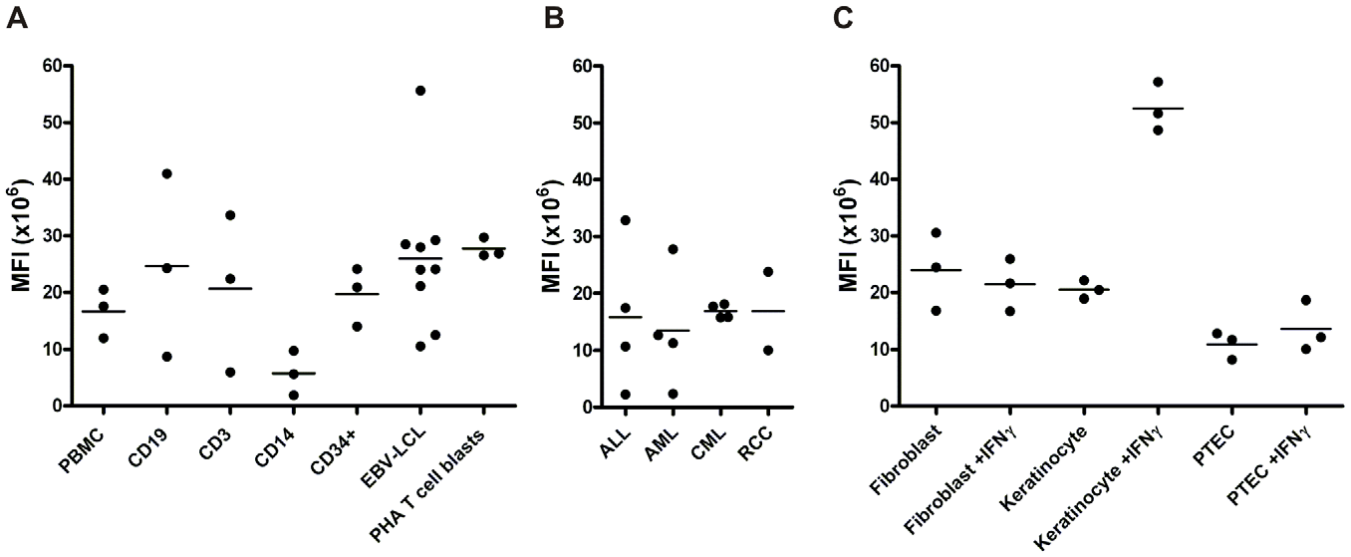
ZNF419 microarray gene expression analysis of (malignant) hematopoietic and non-hematopoietic cells. (**A**) ZNF419 expression of various hematopoietic cell types isolated from PBMC. (**B**) ZNF419 expression of hematopoietic malignancies sorted for CD34, CD33, and CD19/CD34. (**C**) ZNF419 expression of normal tissues +/− IFN*γ*. Data are displayed as MFIx10^6^. 0–10 =  no expression, 10–100 =  low expression, 100–1000 =  intermediate expression, >1000 =  high expression.

Expression of ZNF419 in various (malignant) hematopoietic and non-hematopoietic cell samples was performed by microarray gene expression analysis. Analysis of primary hematopoietic tissues (Figure 5A) show that the overall expression of the gene is low, as is the case for hematopoietic malignancies (Figure 5B). Although fibroblast, keratinocytes, proximal tubular epithelial cells (PTEC) and RCC show low expression levels as well, the keratinocytes show an upregulation of ZNF419 when cultured in the presence of IFN-γ (Figure 5C).

### Detection of ZAPHIR-specific CD8+ T cells in vivo

**Figure 6:**
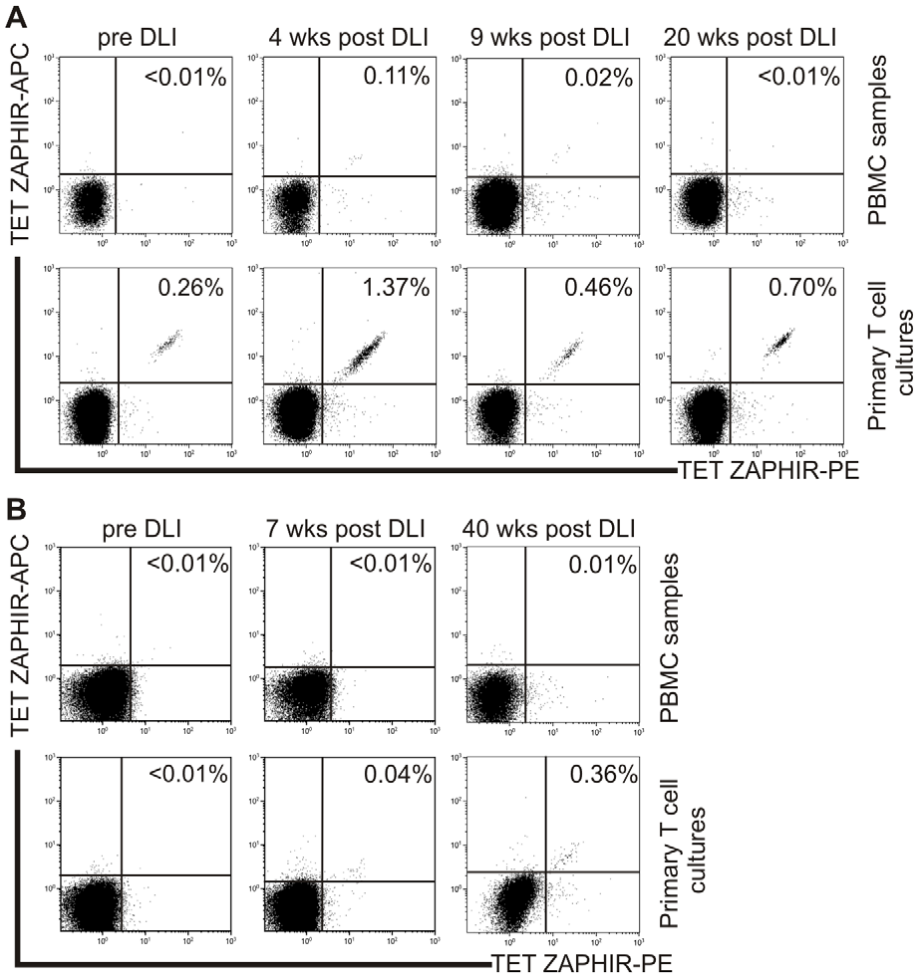
Detection of ZAPHIR-specific CD8+ T cells in peripheral blood of RCC patient UPN686 and CML patient UPN539. (**A**) PBMC collected before DLI and 4, 9, and 20 weeks post DLI were stained with PE- and ACP-conjugated ZAPHIR/HLA-B7 tetramer, CD8 AlexaFluor 700, CD4, CD14, CD16 and CD19 FITC and Sytox Blue. Cells were analyzed by flow cytometry by gating on CD8+FITC-Sytox Blue- lymphocytes. The percentage of tetramer+ cells among CD8+ T cells is depicted. The remaining PBMCs were stimulated once with ZAPHIR peptide pulsed (10 µM) EBV-LCL of the donor and assayed on day 7 for ZAPHIR-tetramer+ CD8+ T cells. (**B**) PBMC collected pre-DLI, 7 and 40 weeks post-DLI were stained and gated as described under (A). The remaining PBMCs were stimulated once with ZAPHIR peptide-pulsed (10 µM) EBV-LCL of the donor and assayed on day 7 for ZAPHIR-tetramer+ CD8+ T cells.

Next, we determined the rate of ZAPHIR disparity in HLA-B7-matched SCT donor-recipient pairs using a competitive allele-specific PCR. In 98 donor-recipient pairs (including RCC patient UPN686), we identified 13 ZAPHIR-positive patients (i.e. 13.3%) that had received a transplant from a ZAPHIR-negative donor. To detect ZAPHIR-specific CD8^+^ T cells in these mismatched patients *in vivo*, we constructed an HLA-B7 tetramer using the 10-mer peptide IPRDSWWVEL. First, we analyzed PBMCs samples of RCC patient UPN686 from which the ZAPHIR-specific CTL B1 was isolated. In addition, specific detection of ZAPHIR-tetramer^+^ CD8+ T cells was assessed after *in vitro* stimulation of PBMC samples with peptide-pulsed donor EBV-LCL. At fourteen weeks post-SCT (i.e. pre-DLI) <0.01% ZAPHIR-tetramer^+^ cells could be directly detected in the PBMC sample, but after stimulation the percentage increased to 0.26% within the total CD8+ T cell population (Figure 6A). Interestingly, the percentage ZAPHIR-tetramer^+^ CD8+ T cells increased at 4 weeks after DLI to a level of 0.11%, and persisted at a low frequency (∼0.01%) up to 20 weeks post-DLI. *In vitro* stimulation resulted in an increase of ZAPHIR-specific CD8+ T cells up to 1.37% for week 4, 0.46% for week 9, and 0.70% for week 20 post-DLI (Figure 6A). These data show that ZAPHIR-specific CD8+ T cells with the capability of targeting RCC metastases emerged in RCC patient UPN686 following SCT and DLI in the absence of GVHD.

Specific detection of ZAPHIR-tetramer^+^ CD8+ T cells was also performed in the peripheral blood of 12 additional ZAPHIR-mismatched patients. In 1 of these 12 patients, chronic myeloid leukemia (CML) patient UPN 539, 0.04% and 0.36% ZAPHIR-tetramer+ CD8+ T cells could be detected in the *in vitro*-stimulated T cell cultures obtained from PBMC samples at 7 and 40 weeks post-therapeutic DLI, while no (<0.01%) tetramer+ cells could be detected directly in the PBMC (Figure 6B). Furthermore, ZAPHIR-specific CTL could be cloned from PBMC collected 40 weeks post-therapeutic DLI (Figure S4). These data indicate that ZAPHIR-specific CD8+ T cell responses can also be detected in transplanted CML patients.

## Discussion

MiHA are considered to play a dominant role in mediating GVT reactivity after HLA-identical allogeneic SCT for both hematological malignancies and solid tumors. Especially MiHA with a hematopoietic- or tumor-restricted distribution are promising targets to boost GVT reactivity without enhancing GVHD. For the development of tumor-specific immunotherapy it is important to enlarge the spectrum of molecularly identified MiHA that are selectively expressed. In this study, we characterized antigens targeted by allogeneic donor T cells in metastatic RCC patients treated with partial T cell-depleted RIC-SCT followed by DLI. Using PBMCs collected from two patients, showing either stable disease or partial response, we succeeded in expanding CD8+ CTLs with the capability of targeting MiHA expressed by RCC cells. Interestingly, a TCR-Vb4+CD8+ CTL targeting a novel HLA-B7-restricted MiHA, designated ZAPHIR, was isolated from RCC patient UPN686 showing partial regression of lung metastases in the absence of GVHD. Flow cytometry-based cytotoxicity assays revealed that ZAPHIR-specific CTL B1 mediated efficient lysis of HLA-B7+ RCC cell lines. In addition, ZAPHIR-specific CTL also mediated cytotoxicity against an HLA-B7+ brain tumor cell line. Furthermore, while EBV-LCL were recognized by ZAPHIR-specific CTL, PHA-stimulated T cells and cytokine-treated bone marrow (BM)-derived fibroblasts did no induce IFN-γ production. These data indicate that the HLA-B7-restricted MiHA ZAPHIR is co-expressed by solid tumors and transformed B cells. Notably, emergence of ZAPHIR-specific CD8+ T cells in RCC patient UPN686 as well as CML patient UPN 539 was observed in the absence of GVHD. Microarray gene expression analysis of several (malignant) hematopoietic cells and normal tissues show that expression of ZNF419 is low. This might explain the absence of an immune response, as is the case *in vivo* in non-malignant tissues, and therefore no GVHD could be observed. However, as the keratinocytes cocultured with IFN*γ* show that under inflammatory conditions ZNF419 is upregulated and could lead to an immune response. Since the tumor tissues are surrounded by an inflammatory environment, a GVT response mediated by MiHA-specific T cells could occur *in vivo*.

In order to identify the novel MiHA recognized by CTL B1, we used the WGAs method that was recently described by Van Bergen et al [16]. This method makes use of the SNP-genotyping of unrelated HLA-B7+ individuals who were divided in a MiHA+ and MiHA- group and yielded a highly specific genomic location on chromosome 19q13.43. This led to the rapid identification of ZNF419 as the gene of interest showing WGAs to be a very suitable method for MiHA identification. Further analysis revealed that the novel ZAPHIR epitope is located on the exon4-intron4 boundary which is the result of a G to A substitution in the splice donor site leading to an alternative spliced ZNF419 variant. The mechanism that MiHA immunogenicity can arise from alternative splicing due to a single intronic SNP, has been previously described for the MiHA ACC-6 [17].

In RCC patient UPN677, we demonstrated emergence of SMCY.A2-specific CD8+ T cells after DLI without the clinical manifestations of GVHD. Interestingly, Hambach et al. showed that SMCY.A2-specific CTL are capable of efficiently targeting solid tumor cells in a three-dimensional micro tumor model [11]. Therefore, our clinical findings suggest that emergence of SMCY.A2 CTL may have played a role in stabilization of the tumor growth after allogeneic SCT and DLI in patient UPN677. Interestingly, high numbers of SMCY.A2 CD8+ T cells up to 3.6% of the total CD8+ T cell population in patient UPN677 did not induce clinical signs of GVHD to either skin, liver or gut. Earlier studies have shown that SMCY is ubiquitously expressed, and SMCY-specific CTL responses have been associated with GVHD [9], [18]–[20]. However, GVHD is a multi-factorial disease process to which many factors contribute, and we speculate that lack of other inflammatory triggers prevented the development of acute GVHD (reviewed by Ferrara et al [21]).

Currently, the role of allogeneic RIC-SCT for the treatment of metastatic RCC patients is still unclear. New developments in treatment of metastatic RCC have significantly changed the treatment strategies for these patients. However, the novel drugs that are currently used do not cure patients and the incidence of complete remissions is low. Therefore, new strategies should continue to be explored and the discovery of antigenic targets of alloreactive T cells in transplanted RCC patients may allow the development of tumor-specific post-transplantation strategies such as vaccination or adoptive T cell transfer. Two earlier studies have identified target antigens at the molecular level using CD8+ CTL isolated from patients with metastatic RCC treated with allogeneic SCT. Tykodi et al. identified an HLA-A*0201-restricted MiHA, which is encoded by the *C19orf48 gene* located on chromosome 19q13 [14]. In addition, recognition of the non-polymorphic RCC over-expressed human endogenous retrovirus type E (HERV-E) antigen by alloreactive T cells was described by Takahashi et al [15]. Not only tumor-specific MiHA are interesting for these purposes, but also hematopoietic-restricted MiHA with aberrant expression in solid tumor cells may be useful for this strategy. Genotype frequency analysis in HLA-B7 allo-SCT couples revealed a disparity rate of 13.3%, making the ZAPHIR MiHA therapeutically relevant as is the case for the other, commonly mismatched MiHA HA-1, LRH-1 and BCL2A1 [22]–[24]. Adoptive transfer of a single dose of HA-1 CTLs was effective in eradicating disseminated solid tumors in a mouse model [11]. Therefore, adoptive immunotherapy with *ex vivo*-generated CTLs directed against MiHA expressed by RCC might be an attractive adjuvant approach as has also been studied in patients with leukemia [25]. Alternatively, patients could be vaccinated with peptides or DCs loaded with peptides or electroporated with MiHA-encoding mRNA [26], [27].

In conclusion, this study describes T cell responses after partial T cell-depleted RIC-SCT followed by DLI in patients with metastatic RCC. Our transplant procedure resulted in stable engraftment, manageable GVHD and T cell responses in two patients. SMCY-specific CD8+ T cells were identified in a patient with stable disease, and CD8+ T cells targeting the novel HLA-B7-restricted MiHA ZAPHIR in the second patient with a partial response. Although the role of allogeneic RIC-SCT in RCC may be limited in the near future, this study shows that the approach of partial T cell-depleted SCT followed by DLI induces MiHA-specific T cell responses potentially targeting RCC tumor cells. The significance for cellular immunotherapy of the novel MiHA ZAPHIR is demonstrated by the emergence of ZAPHIR-specific CTL after allo-SCT, and its *ex vivo* cytotoxic activity against RCC and transformed B cell lines. Furthermore, identification of the antigenic targets of alloreactive T cells remains important for the further understanding of the GVT response in different malignancies and for development of strategies to selectively target tumor cells after allogeneic SCT.

## Materials and Methods

### Patient eligibility

From December 2002 till August 2003, four consecutive patients with metastatic RCC have been treated in our transplantation program. The eligibility criteria for patients included histological proven, clinically progressive metastatic RCC, failure on earlier cytokine based systemic therapy and no other curative treatment available. This study was approved by the institutional review board of RUNMC, and all patients gave written informed consent.

### Conditioning regimen, GVHD prophylaxis and DLI

Patients received partial T cell-depleted RIC-SCT. The conditioning regimen consisted of total lymph node irradiation on each of three consecutive days followed by cyclophosphamide 50 mg/kg body weight intravenously on each of four consecutive days (total dose 200 mg/kg bodyweight). Inguinal and femoral lymph node regions were also irradiated. A total dose of 12 Gy was delivered in 2 Gy fractions, twice daily, on three consecutive days.

Donors were HLA-identical siblings. Following T cell depletion, CD3+ T cells were added back to generate a stem cell graft containing a fixed number of 0.5×10^6^ T cells/kg body weight of recipient. All patients received CsA 3 mg/kg/day by continuous intravenous infusion from days -1 to +14. Thereafter CsA dose was reduced to 2 mg/kg/day and continued until day 21, when CsA was tapered off for 8–10 weeks.

Patients underwent CT-scan of abdomen and chest before, and every 3 months after RIC-SCT to assess disease response. Patients without acute GVHD grade >II, without chronic GVHD and with persistent disease received DLI four weeks after discontinuation of immunosuppression. If no GVHD occurred and residual disease persisted, a second DLI was administered two months later. The first DLI dose consisted of 0.1×10^8^ T cells/kg bodyweight of the recipient, and the second DLI dose was 0.7×10^8^ T cells/kg.

### Cell isolation and culture

CD8+ CTL lines H and B were isolated from PBMC obtained one and three months after DLI-1, respectively, by weekly stimulation with PBMC obtained before SCT in Iscove's modified Dulbecco's medium (IMDM; Invitrogen, Carlsbad, CA) supplemented with 10% human serum (HS; Sanquin blood bank, Nijmegen, the Netherlands). After initial stimulation, CTL line H, CTL line B, the HLA-B7 alloreactive CTL Kor18 and the HLA-A2 alloreactive CTL 1E2 (0.5*10^6^) were cultured in IMDM/10% HS containing irradiated (80 Gy) recipient EBV-LCL (0.5*10^6^), irradiated (60 Gy) allogeneic PBMC (0.5*10^6^) from two donors, 100 IU/ml IL-2 (Chiron, Emeryville, CA) and 1 µg/ml PHA-M (Boehringer, Alkmaar, the Netherlands). All cell lines and primary cells were cultured in IMDM/10% fetal calf serum (FCS; Integro, Zaandam, The Netherlands). Fibroblasts were cultured from BM aspirates obtained from healthy stem cell donors. BM was resuspended in 20 ml IMDM/20% FCS and incubated overnight at 37°C in tissue culture flasks. The non-adherent fraction was removed and fibroblasts were further cultured in IMDM/20% FCS to passage 3 before analysis. T cell blasts were generated by stimulating PBMC in IMDM/10% HS with 20 µg/ml PHA-M for 3 days. Thereafter, PHA-activated T cells were washed and further cultured with 100 IU/ml IL-2 for 2 additional days.

### IFN-γ secretion assay

IFN-γ producing CTLs were detected and isolated using the IFN-γ secretion assay (Miltenyi Biotec). Briefly, 1×10^6^ CTLs were incubated with 1×10^6^ irradiated (30 Gy) recipient EBV-LCL in a total volume of 2 ml IMDM/10% HS. After 16 hours of incubation at 37°C, cells were harvested, washed with PBS/0.5% FCS and 5 mM EDTA, and labeled at a concentration of 10^8^ cells/ml with 50 µg/ml Ab-Ab conjugates directed against CD45 and IFN-γ for 10 minutes on ice. Subsequently, cells were diluted with IMDM/10% FCS at 1×10^5^ cells/ml and allowed to secrete IFN-γ for 45 minutes at 37°C. After the cytokine-capturing period, cells were collected, resuspended at a concentration of 10^8^ cells/ml in PBS/0.5% FCS/5 mM EDTA, and stained with 5 µg/ml PE–conjugated anti-IFN-γ mAb and FITC-conjugated CD8 mAb for 20 minutes at 4°C. Finally, cells were analyzed and sorted using an Epics Elite flow cytometer (Beckman Coulter).

### Flow cytometry

PE- and APC-labeled SMCY.A2 tetramers containing peptide FIDSYICQV and ZAPHIR.B7 tetramers containing peptide IPRDSWWVE were produced as described previously [28]. PE-labeled tetramer for the HLA-A2-restricted EBV peptide GLCTLVAML were purchased from Beckman Coulter. PBMC or CTL lines were incubated with 20 µg/ml tetramer for 15 min at room temperature. After washing with PBS/0.5% bovine serum albumin (BSA; Sigma,St Louis, MO, USA), cells were labeled with AlexaFluor700-conjugated anti-CD8 (Invitrogen) in combination with FITC-conjugated CD4, CD14, CD16 and CD19 (Beckman Coulter) for 30 min at 4°C. Finally, cells were washed and resuspended in PBS/0.5% BSA containing 0.2 µM Sytox Blue (Invitrogen) marking dead cells and analyzed using the Cyan flow cytometer (Beckman Coulter). Tetramer staining using both APC and PE showed double positive events, allowing optimal discrimination between background staining and positive cells. All FITC-positive cells were gated into a dump channel and played no role in further analysis.

### Retroviral transduction of HLA-B*0702 in cell lines and CTL stimulation assay

LZRS-HLA-B*0702-IRES-EGFP vector was used to generate a stable producer cell line. Retroviral transduction was performed using non-tissue culture-treated 35-mm^2^ dishes (Becton Dickinson) coated with 10 µg/ml retronectin (Takara Biomedicals, Shiga, Japan). In brief, 10^6^ target cells were resuspended in 2 ml virus supernatant and transferred to retronectin-coated dishes. After 24 h of incubation, cells were collected and incubated with fresh virus supernatant and used in CTL stimulation assays [29]. Release of IFNγ was determined by ELISA (Pierce Endogen, Rockford, IL).

### Flow cytometry-based cytotoxicity studies

Flow cytometry-based cytotoxicity assays were performed as previously described [30]. HLA-B7^+^ cell lines were labeled with 2.5 µM carboxyfluorescein diacetate succimidyl ester (CFSE; Molecular Probes Europe). Target cells (1×10^4^) were co-cultured with unlabelled effector cells (3×10^4^) at an E:T ratio of 3∶1 in a total volume of 200 µl IMDM/10% FCS containing 25 U/ml IL-2 in 96-wells flat-bottom plates. After 1–3 days of co-culture, cells were harvested and 7-amino-actinomycin D (7AAD; Sigma-Aldrich, St Louis, MO) was added. Numbers of viable target cells were quantified by flow cytometry.

### Whole Genome Association scanning (WGAs)

A panel of 87 HLA-B*0702 expressing EBV-LCL was selected to perform WGAs. To test recognition of the EBV-LCL panel by the T cell clone, triplicate samples of 6×10^4^ EBV-LCL were thawed, washed and cultured for 2 days, and 5×10^3^ MiHA specific T cells were added to each well. After 24 hours, culture supernatant was used for IFN-γ ELISA. Of each EBV-LCL, PCR-free whole genome amplification was performed and analyzed by Illumina Human1M-duo arrays containing probes for 1.1 million SNPs (Illumina, San Diego, CA) [31]. EBV-LCL panel was divided into MiHA^pos^ and MiHA^neg^ groups using 5 times the level of IFN-γ production in the absence of EBV-LCL as a threshold for recognition. WGAs was performed by combining T cell recognition with SNP-genotyping data. The level of matching between both patterns was calculated according to Fisher's exact test.

### Genotyping using KASPar system

Genotyping of the HLA-B7-matched SCT donor-recipient pairs was conducted by KASPar (KBioscience, Herts, UK), a fluorescence-based competitive allele specific PCR which utilizes non-labeled primers. Details of the process and primer sequence of SNP rs2074071 can be obtained from KBioscience.

### Microarray gene expression analysis

Total RNA was isolated from various (malignant) hematopoietic and non-hematopoietic cell samples using small and micro scale RNAqueous isolation kits (Ambion, Inc., Austin, TX). Malignant hematopoietic cell samples included CML, AML and ALL cells isolated by flow cytometry based on expression of CD34, CD33, and CD19/CD34, respectively. Non-malignant hematopoietic cells included total PBMC, and B-cells, T-cells and monocytes isolated from total PBMC by flow cytometry based on expression of CD19, CD3 and CD14, respectively. In addition, hematopoietic stem cells were isolated from G-CSF-mobilized peripheral blood by flow cytometry based on expression of CD34. Non-hematopoietic cell samples included cultured skin-derived fibroblasts, keratinocytes and PTEC (kindly provided by Dr. C. van Kooten, Leiden, Netherlands). Finally, total RNA was isolated from EBV-B cells, PHA-T blasts and RCC cell lines. (kindly provided by Dr. E.M.E. Verdegaal, Leiden, Netherlands). RNA samples were amplified for microarray gene expression analysis using the Illumina TotalPrep RNA amplification kit (Ambion). Briefly, total RNA was treated with DNase I using the TURBO DNA-*free* kit (Ambion), and converted to cDNA by reverse transcription. After purification of cDNA, cRNA was synthesized by in vitro transcription. cRNA samples were prepared following the whole-genome gene expression direct hybridization assay (Illumina, Inc., San Diego, CA), and resuspended cRNA samples were dispensed onto Human HT-12 v3 Expression BeadChips (Illumina). Hybridization was performed in the Illumina hybridization oven for 17 hours at 58°C. Microarray gene expression data were analyzed using Rosetta Resolver 7.2 gene expression data analysis system.

### Construction of full-length and truncated ZNF419 constructs

The full-length ZNF419 transcript in pCMV6-XL was obtained by Origene, Rockville, MD (sc 316397). The different splice variants of the ZNF419 transcript containing exon 1 to 5 and the exon 4/intron 4 boundary were generated by RT-PCR of the MiHA+ recipient and MiHA- donor EBV-LCL. The following primers were used to amplify ZNF419 transcript variants: ZNF419-F 5′-AAAGGTACCTCTACTCACAGGCTCCGATGG-3′ and ZNF419-R 5′-AAACCTAGGGAGGTTGAAACGCTGGCTAAAG-3′. These primers contained BamHI and KPNI digestion sites. PCR products were purified from 1.5% agarose gel using the Qiagen gel extraction kit, and cloned into vector pcDNA3.1(+) (Invitrogen) using the BamHI and KPNI digestions. Constructs were verified by sequencing, and used for transfection into 293T-HLA-B*0702/ICAM/CD80 cells. In brief, 3*10^4^ 293T-HLA-B*0702/ICAM/CD80 cells were plated into poly-D-lysine coated 96-wells flat bottom wells (Becton Dickinson, Franklin Lakes, NY), cultured overnight at 37°C, and then transfected with 200 ng plasmid DNA using lipofectamin reagent (Invitrogen). Transfected cells were cultured for 2 additional days and used in CTL stimulation assays.

### Epitope prediction and epitope reconstitution assay

HLA-B7 binding peptides were predicted using the NetMHC 3.2 server (http://www.cbs.dtu.dk/services/NetMHC/). Peptides were synthesized by Proimmune and dissolved in DMSO. Peptide stock solutions were diluted in IMDM to a concentration of 1 mM. In peptide recognition assays, target cells were preincubated with peptide for 1 hour in a volume of 1 ml prior to the addition of CTL.

## Supporting Information

Figure S1 **Generation of MiHA-reactive CD8+ CLT lines from patients with RCC after allogeneic RIC-SCT.** (**A**) Detection of IFN-γ secreting CD8+ T cells in T cell lines generated from patient UPN677 and UPN686 after co-culture with EBV-LCL of the recipient. Stimulated T cells were stained with PE-conjugated IFN-γ detection reagent and FITC-conjugated anti-CD8 mAb, and analyzed by flow cytometry. (**B**) Production of IFN-γ by CTL lines H and B upon stimulation with recipient (Rt) EBV-LCL, donor (Do) EBV-LCL or medium. Data are displayed as mean IFN-γ release ± SD of triplicate wells.
(TIF)

Figure S2 **WGAs using 87 HLA-B7 positive EBV-LCL revealed a cluster of 5 SNPs to associate on chromosome 19 cluster of SNP.** (**A**) Detection of IFN-γ-secretion by CTL B1, here depicted as OD450, upon stimulation with 87 HLA-B7+ EBV-LCL showed 42 EBV-LCL to be recognized. The threshold for recognition was set at 5 times the level of IFN-γ production in the absence of EBV-LCL. (**B**) Detail of chromosome 19 showing all SNP that cluster according to Fisher's exact test. No other SNP cluster was found at any other chromosome.
(TIF)

Figure S3 **ZNF419 splice variant.** (**A–B**) The full length transcript of ZNF419 (NM_001098491) consists of 5 exons which are spliced into an additional 6 splice variants (NM001098492 through NM_001098496 and NM_024691). Due to SNP rs2074074 in the splice donor site an additional transcript occurs in patient UPN686 which includes exon 4 (grey) leading to a stop-codon (*). (**C**) The different splice variants that were expressed by patient UPN686 and its related donor were amplified as a pool, using the primerset as depicted (→) and cloned into pcDNA3.1(+).
(TIF)

Figure S4 **Generation of ZAPHIR-reactive CD8+ CTL Lines from CML patient UPN539 40 weeks post DLI.** (**A**) PBMC collected 40 weeks post DLI were sorted for TET+CD8+ T cells (inside square) and cloned into single cells. (**B**) Production of IFN-γ by ZAPHIR-specific CTL clones with ZAPHIR-peptide pulsed donor EBV-LCL, donor EBV-LCL or medium. Data are displayed as mean IFN-γ release ± SD of triplicate wells.
(TIF)
